# Research on the Relationship of Consumption Emotion, Experiential Marketing, and Revisit Intention in Cultural Tourism Cities: A Case Study

**DOI:** 10.3389/fpsyg.2022.894376

**Published:** 2022-07-13

**Authors:** Hu Chen, Yingchao Wang, Na Li

**Affiliations:** ^1^School of Public Administration, Shandong Agricultural University, Tai'an, China; ^2^School of Civil Engineering and Architecture, University of Jinan, Jinan, China; ^3^Department of Economics, Sejong University, Seoul, South Korea

**Keywords:** cultural tourism cities, experiential marketing, revisit intention, structural equation model, consumption emotion

## Abstract

Experience marketing plays an important role in improving the quality and upgrading tourism services in cultural tourism cities and helps guide the planning and development, commodity design, and business management of cultural tourism products. However, the urgent problems that need to be solved are as follows: How does experiential marketing in cultural tourism cities affect tourists' consumption behavior? How to adjust consumption emotion in tourist experience and revisit intention? Starting from the experience needs of tourists, this study selected Jinan city, represented by “Qilu culture,” as the research object; discussed the relationship between experiential marketing, consumption emotion, and revisit intention; and used a structural equation model to verify the relationship between the three. This study divided the perception of experiential marketing into four dimensions—sensory experience, action experience, emotional experience, and thinking experience, and divided tourists' revisit intention into two dimensions— “revisit” and “recommendation”. Totally, 305 tourists were randomly selected to participate in the questionnaire survey, and they came from 34 provinces in China. The results showed that cultural tourism cities can enhance tourists' positive consumption emotion through experiential marketing over time, and high-quality tourist consumption experience directly motivates tourists to revisit intention and then promotes tourists to go to cultural tourism cities for secondary consumption. These results suggest that cultural tourism cities should start from Maslow's hierarchy of needs theory, pay attention to the needs of tourists at different levels, and adopt effective experiential marketing strategies from tourism experience to improve the quality of tourists' travel experience and promote tourists' revisit intention.

## Introduction

After agricultural economy, commodity economy, and service economy, experience economy is the fourth economic stage of world economic development (Gunter and Kenny, 2021). Experience economy has evolved from the separation of service economy. It pursues the degree of customer satisfaction and, at the same time, pays attention to the self-experience of customers in the process of consumption. As early as 1979, Erik Cohen put forward the concept of tourism experience, and he believed that tourism experience is an emotional process (An and Alarcón, 2021). What Mariana and colleagues consider memorable travel experiences seem to be closer to the experience economy of Pine and Gilmore, which has influenced research in the field of tourism (Coelho et al., 2018). Bongkoo Lee believes that the ultimate goal of leisure tourism is usually to obtain high-quality tourism experience, and the ultimate goal of tourism destinations and their managers is to provide tourists with a pleasant tourism experience (Lee, 2021).

In the experience economy, consumers' process of consuming products is a process of experiencing services. This experience is not only unique but also multi-dimensional. A good experience or a bad experience will leave an indelible memory in consumers' mind, especially customers' first consumption experience will be unforgettable. Destination marketers and managers can develop programs of experience marketing to increase the onsite experience of tourism destination and to strengthen tourists' loyalty intentions (Rather and Hollebeek, 2019, 2020). People's emotions during leisure travel will directly affect tourists' consumption behavior and willingness (Lo and Wu, 2014; Liu et al., 2021). As customers age, customer experience has an increasing impact on behavioral intent (Rather and Hollebeek, 2021). The impact of customer engagement on revisit intent was most pronounced where customer experience and co-creation were elevated (Rather et al., 2021). In the same place, personal experience will produce positive and negative emotions; positive emotions include surprise, comfort, pleasure, and impression, and negative emotions include disappointment, boredom, unhappiness, and anger (Han et al., 2010; Hou et al., 2013; Min et al., 2018). The revisit invention of tourists refers to the invention of tourists to participate again after visiting or participating in a certain tourism activity, mainly including “revisit” and “recommendation”. For tourist attractions, the most direct manifestation of tourists' loyalty is their inventions to visit the cities again and recommend them to others.

To promote the integration and development of world culture and tourism, this study takes the experience needs of tourists as the starting point and selects Jinan city, which is represented by “Qilu culture,” as the research object. This study divides the perception of experiential marketing into four dimensions—sensory experience, action experience, emotional experience, and thinking experience, and divides tourists' revisit intentions into two dimensions: “revisit” and “recommendation”. This study explores the relationship between experiential marketing, consumption emotion, and revisit intention and uses the structural equation model to verify the relationship between the three.

## Literature Review

Experiential marketing is the main research content of tourism experience, which was proposed by American Pine and Gilmore in 1998. According to Maslow's hierarchy of needs, the experience of consumers before, during, and after consumption is both rational and emotional, and this experience plays a crucial role in the study of consumer behavior and corporate brand management. Chen examined the relationship among tourists' experience quality, perceived value, satisfaction, and behavioral intentions, revealing the direct impact of experience quality on perceived value and satisfaction (Ge et al., 2021). Drengner, Gaus, and Jahn believe that experiential marketing is one of the feasible methods to arouse consumers' latent demand for products (Drengner et al., 2008). Dholakia and Zhao believe that through experiential marketing, products can not only trigger consumers' impulse purchases but also affect their subsequent purchase intentions (Meilatinova, 2021). Schmitt put forward the five modules of experiential marketing in terms of the measurement dimension: sense, feel, act, think, and relate (Schmitt, 1999). In addition to Schmitt's research, Brakus explored the four dimensions of experiential marketing from the perspective of brand perception: sensory experience, affective experience, intellectual experience, and behavioral experience (Brakus et al., 2009).

Consumption emotion of tourists is not only an important part of the tourism experience but also a direct product of experience marketing. Consumption emotion generated by each tourist in the travel process will directly affect the tourists' consumption behavior and the willingness of secondary consumption. Holbrook MB studied the mediating role of emotion in the game product experience process (Lester et al., 2021). Oliver detected the emotions of customers when enjoying service packages (Mano et al., 2019). Ladhari updated the influence of emotion on satisfaction through a study of customers' experience with movies (Ladhari, 2007). Allen, Machleit, and Kleine believed that it is particularly important to understand consumption emotion under various consumption situations because consumption emotion is an important part of customer response (Kheirabadi et al., 2019). (Liu et al., 2021) studied the impact of consumer sentiment on consumers' electronic word-of-mouth (eWOM) behavior and emotional preferences. Rather (2017, 2018), Rather et al. (2019), and Köchling (2021) explored the relationship between social proof and experiential marketing constructs, reflecting the relationship between customer brand identity and satisfaction, trust, commitment, and its impact on hotel brand loyalty.

The concept of revisit intention originated from the concept of repurchase intention, and it was not applied to tourism field until 1989. Gyte and Phelps studied the behavior of British tourists to Spain and found that tourists have a strong revisit intention (Gyte and Phelps, 1989). Kozak believes that revisit intention is the willingness of tourists to agree to visit a certain destination or other attractions in the same country again (Kozak, 2003). Bigne, Sanchez, and Sanchez believed that behavioral intention includes recommendation to others and positive word of mouth (Bigné et al., 2005). It can be seen that more and more scholars have adopted “revisit” and “recommendation” as the measurement dimensions of tourists' revisit intention tourism (Rodríguez Molina et al., 2012; Chung and Petrick, 2013; Todorovic et al., 2017; Rojas-De-Gracia and Alarcon-Urbistondo, 2019; Marques et al., 2021).

To sum up, in the process of tourism experience, tourists will have many types of tourism emotions. Research studies on consumption emotion at home and abroad are mainly conducted from the perspective of emotional cognition, with a wide range of directions, especially empirical research. Many experts have also developed different consumption emotion scale indicators and verified them through empirical evidence. At present, most of the pieces of empirical evidence are applied to the service industry, catering industry, hotel industry, tourism factory, theme park tourism, etc., while the emotional research on cultural tourism cities at home and abroad is still insufficient. Therefore, it is worth exploring whether tourists' consumption emotion has an impact on tourists' consumption or revisit intention in cultural tourism cities.

## Theoretical Model and Research Design

### Case Study of Cultural Tourism City—Jinan City

Under the background of experience economy, the purpose of tourists' travel is to obtain the desired experience, so paying attention to experience marketing has become the core content of the service and management of tourism enterprises. According to Schmitt's experiential marketing theory, experience must have a “theme,” and experiential marketing should start from and serve this theme. Jinan city is a cultural tourism city with the theme of “Qilu culture,” with thousands of years of historical and cultural accumulation, which meets the needs of this research. Therefore, this study selects tourists from Jinan as the research object.

“Qilu culture” is the collective name of “Qi culture” and “Lu culture.” The “Qilu culture” proposed the Taoist theory represented by Jiang Taigong, and absorbed the local indigenous culture (Dongyi culture) and developed it. There are differences between the two ancient cultures. Relatively speaking, “Qi culture” is utilitarian, while “Lu culture” emphasizes ethics, “Qi culture” emphasizes innovation, and “Lu culture” respects tradition. The two cultures have gradually and organically merged together in the development, forming the “Qilu culture” with rich historical connotations.

Jinan city, Shandong Province, China, also known as “spring city,” is one of the “top 10 best living cities in China” in 2020–2021. Jinan is a national historical and cultural city, an excellent tourist city in China, and an important part of Shandong tourism “one mountain, one water, and one saint,” attracting many domestic and foreign tourists every year. In 2021, it received 100.26 million domestic and foreign tourists, an increase of 8.5% over 2020. Among them, 99.803 million domestic tourists were received, an increase of 8.5%; 457,000 inbound tourists were received, an increase of 12.2%. There are one 5A-level scenic spot, 17 4A-level scenic spots, and two tourist resorts above the provincial level. Jinan's tourism culture highlights the characteristics of “Qilu culture,” and there are four major spring groups: Baotu Spring, Black Tiger Spring, Pearl Spring, and Wulong Pool.

### Theoretical Model

Cultural tourism cities mainly focus on characteristic cultural experiences, sightseeing factories focus on product marketing experience, and theme parks focus on clear theme experiences (Rodríguez Molina et al., 2012). Therefore, the relationship between the consumption emotion and the revisit invention generated by the characteristic cultural experience of a cultural tourism city is quite different from that of sightseeing factories and theme parks (Chung and Petrick, 2013).

**Figure 1:**
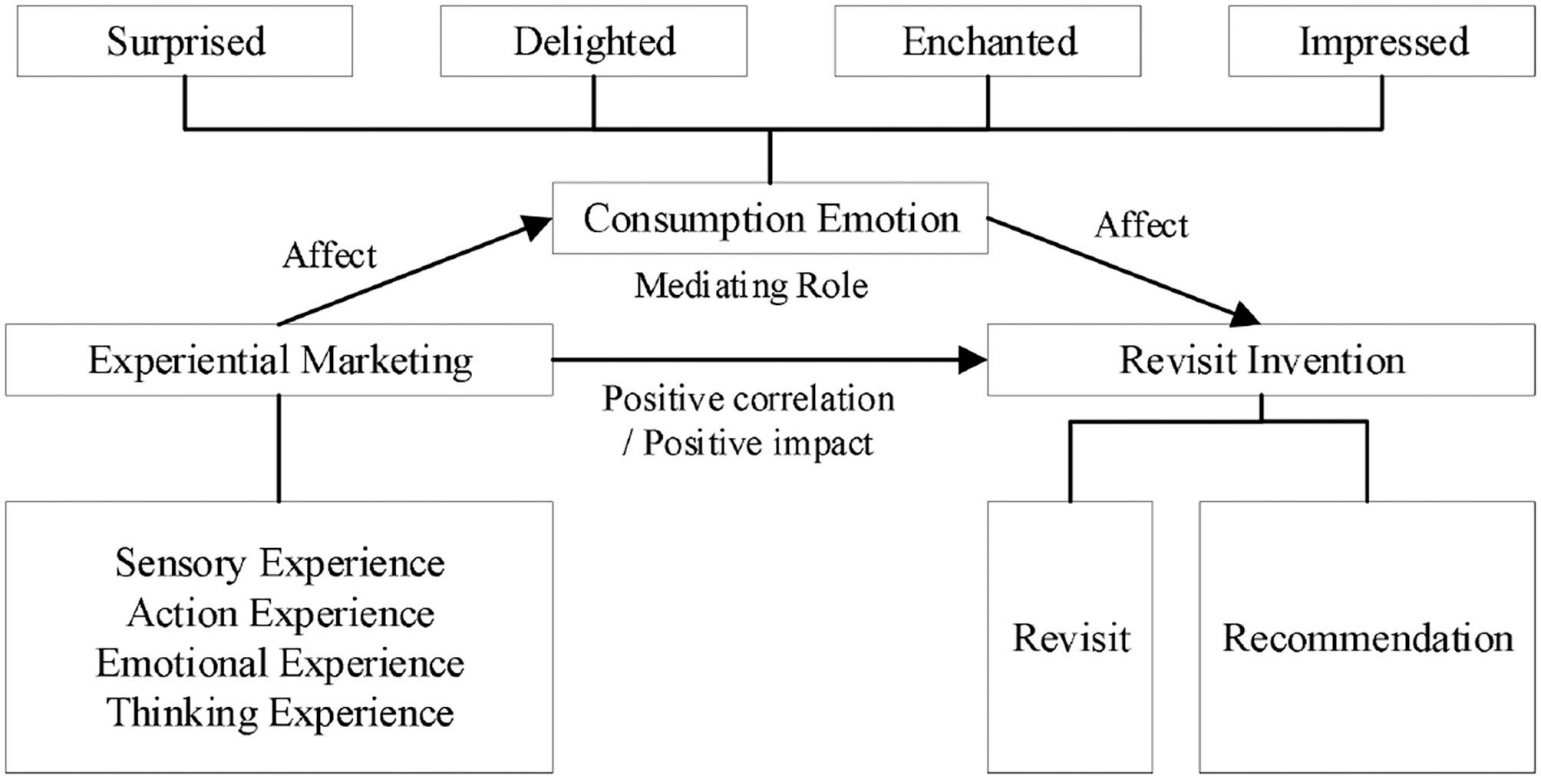
Theoretical model chart.

The purpose of experiential marketing of cultural tourism cities is to allow tourists to have a better consumption experience in the process of tourism. This positive experience will make tourists recommend to friends or family around them, and further drive tourists to revisit again, thus increasing the number of tourists and consumption profits in cultural tourism cities. Experiential marketing can be divided into four categories: sensory experience, action experience, emotional experience, and thinking experience, all of which will have a positive impact on tourists' revisit invention (Han et al., 2010; Liu et al., 2021). For example, the decoration, food or some concerts, and impromptu performances of cultural tourism cities will make a deep impression on tourists, which in turn affects their revisit invention. In between, tourists' consumption emotion will directly affect their revisit invention, such as surprise, delight, fascination, and impression (Lee and Lee, 2016). The theoretical research model using consumption emotion as the mediation is shown in Figure 1.

### Research Hypotheses

Several research hypotheses are proposed, and the problems to be solved are as follows: Does experiential marketing of tourist cultural blocks affect tourists' consumption emotion? Does it affect tourists' revisit invention? Does tourists' consumption emotion affect tourists' revisit invention? Is there a mediating role for tourists' consumption emotion? This study proposes research hypotheses among experiential marketing, consumption emotion, and revisit intention.

#### The Relationship Between Experiential Marketing and Consumption Emotion

A good fashion experiential marketing could attract the consumers before consuming and enhance the experience strength after consuming (Lin, 2009). Tsai (2016) believes that experiential marketing can enhance the overall consumer experience, combining consumers' sensory, emotional, social, and intellectual experiences in new and positive ways. When consumers see weaknesses in their lives reflected in consumer products, they respond positively (Paharia et al., 2011; Shobeiri et al., 2013). This study draws on the four dimensions of Schmitt experiential marketing: sensory, action, emotion, and thinking marketing. Consumption emotion is based on the four emotions of Ignacio Rodrguez del Bosque Hector and San Martin Robert J. Kwortnik's positive consumption emotion scale: surprised, delighted, enchanted, and impressed. Combined with the consumption emotion characteristics of tourists in cultural tourism cities, the following hypothesis is put forward:

***H*_1_**: Experiential marketing positively and significantly affects tourists' consumption emotion.

#### The Relationship Between Consumption Emotion and Revisit Invention

When consumers have positive experience value, there will be higher customer satisfaction, which will have a positive impact on purchase intention (Richins, 1997; Phillips and Baumgartner, 2002). Choi and Kown (2012) believes that the consumption experience of branded coffee shops has a significant impact on positive consumption emotion, as well as revisit invention. Higher customer satisfaction is part of the mediator of the effect of positive consumption emotion on revisit invention and word-of-mouth intentions (Park and Jun, 2010; Jani and Han, 2013). At the same time, the product experience and destination credibility of cultural tourism cities can affect tourists' destination attachment, advocacy, and brand loyalty to cultural tourism cities (Ahmad et al., 2020). This study adopts Ali E. Akgüna's revisit intention dimension (Kwortnik and Ross, 2007; Akgün et al., 2020), divides tourists' revisit intention into two dimensions—“revisit” and “recommendation,” and makes the following hypothesis:

***H*_2_**: Tourists' positive consumption emotion positively and significantly affects tourists' revisit invention.

#### The Relationship Between Experiential Marketing and Revisit Invention

In the experience economy, consumers seek not only products and services but also more enjoyment and fun (Tsai, 2016). There is no doubt that the better the experience of tourists in a place, the stronger their revisit invention (Drengner et al., 2008). Kwon and Park (2012) investigated the impact of experiential marketing on exhibition visitor satisfaction and behavioral intentions. Feeling marketing has no effect on visitor satisfaction, while behavior, association, and thinking marketing have a significant impact on visitor satisfaction. In addition, visitor satisfaction significantly affects visitors' recommendation and revisit invention (Kim, 2008; Tan, 2016). The indirect effects of experiential marketing and customer satisfaction on revisit invention are independent and significant, but the indirect effect of experiential marketing through intermediary customer satisfaction is not significant (Lim and Ahn, 2012; Jeon, 2013; Nugraha, 2019). Tourism experience positively affects tourists' place cognition and attachment emotions and further affects tourists' revisit invention. This study puts forward the following hypothesis:

***H*_3_**: Experience marketing positively and significantly affects tourists' revisit invention.

#### Consumption Emotion Mediates Between Experiential Marketing and Revisit Invention

The mediation hypothesis is a hypothesis that assumes that the effect of an independent variable on the dependent variable is adjusted by the process of the mediating variable, while the independent variable may still affect the dependent variable (Baron and Kenny, 1986; Shoemaker and Lewis, 1999). If the tourist destination managers understand the consumption emotion of tourists and solve their consumption needs, they can achieve the purpose of publicity through the word-of-mouth of these tourists and increase the number of tourists (Enrique Bigné et al., 2008; Song and Qu, 2017). Both positive and negative emotions have significant effects on customer satisfaction and revisit intention. In addition, revisit intention was considered to be a positive function of satisfaction (Jeong, 2012). Chen and Lin (2018) used multiple regression to verify that sensory experience is an important antecedent of purchasing behavior. Certain sensory experiences can significantly affect emotions, and emotions also play a mediating role in the relationship between sensory experience and revisit invention (Jeun and Lee, 2012). Experience value has a mediating relationship between experiential marketing and revisit invention (Shah et al., 2018). This study puts forward the following hypothesis as follows:

***H*_4_**: Tourists' consumption emotion mediates between experiential marketing and revisit invention.

### Variable Measurement

#### Independent Variable: Tourist Experience

The scales related to experience marketing in this study mainly refer to Schmitt's five-module theory and Shen Pengyi's mature scale (Schmitt, 1999; Shen, 2012). The specific content range is as follows: The sensory experience includes the visitor's vision, hearing, taste, smell, and touch. Vision includes architectural style, cultural symbols, structure, and order. Hearing includes background music. The taste includes the cuisine of the scenic area. Emotional experience includes emotions. A distinction needs to be made between emotional experience and consumption emotion. The emotional experience in experiential marketing refers to whether tourists enjoy the process of playing. Consumption emotion refers to whether tourists are willing to spend in the travel process (Derbaix and Pham, 1991; Bußwolder et al., 2019). Action experiences can be generated through bodily sensations, behavioral patterns, lifestyles, and interactions. Thinking experience can be generated through novel activities or cultural stimuli in the cultural tourism city, which can attract tourists' attention and create a sense of wonder, can bring inspiration to them. The specific question design is shown in Table 1.

**Table 1:** Tourist experience measurement questions.

**Visitor experience variables**	**Visitor experience measurement questions**	**References**
Sensory experience	1. ***Jinan*** has unique architectural landscape	
	2. ***Jinan*** has complete tourism facilities	Schmitt, 1999
	3. ***Jinan*** has convenient transportation	Shen, 2012
	4. ***Jinan*** has good tourism services	
Action experience	5. Traveling in ***Jinan*** is a matter of course	Schmitt, 1999
	6. The service staff recommends me to try new things in ***Jinan***	Shen, 2012
	7. ***Jinan*** provides enough consultation	
	8. Interact with folk craftsmen in ***Jinan***	Brakus et al., 2009
Emotional experience	9. ***Jinan*** makes me feel fresh and relaxed	Schmitt, 1999
	10. ***Jinan*** makes me feel warm and cordial	Shen, 2012
	11. The atmosphere of ***Jinan*** makes me want to play	
Thinking experience	12. ***Jinan*** gives me a thought-provoking experience	Schmitt, 1999
	13. ***Jinan*** arouses my curiosity	Shen, 2012
	14. Traveling in ***Jinan*** makes me resonate with “Qilu Culture”	
	15. Traveling in ***Jinan*** gives me a high-quality sense of identity	

#### Intermediate Variable: Consumption Emotion

This study uses the most significant positive consumption emotion when tourists travel to conduct a questionnaire survey on tourists' consumption emotion, including surprise, delighted, enchanted, and impressed. The specific measurement items are shown in Table 2.

**Table 2:** Consumption emotion measurement questions.

**Consumption emotion variable**	**Consumption emotion measurement questions**	**Reference**
Positive consumption emotion	16. I was surprised to play in ***Jinan***	Bosque and Martín, 2008
	17. I was delighted to play in ***Jinan***	
	18. ***Jinan*** enchanted me	
	19. ***Jinan*** impressed me	

#### Dependent Variable: Revisit Invention

Ali E. Akgüna analyzes the relationship between nostalgia, destination image, and tourist behavior (“revisit” and “recommendation”). This study measures the revisit invention from the two dimensions of “revisit” and “recommendation.” The specific measurement items are shown in Table 3.

**Table 3:** Revisit invention measurement questions.

**Revisit invention variable**	**Revisit invention measurement questions**	**Reference**
Revisit invention	20. I will come back to ***Jinan*** soon	Akgün et al., 2020
	21. ***Jinan*** is my first choice for understanding “Qilu Culture”	
Recommendation invention	22. I would recommend ***Jinan*** to anyone who wants to know about “Qilu Culture”	
	23. I will encourage family and friends to visit ***Jinan***	

### Questionnaire Design

The control variables of this study are individual factors of tourists, including gender, age, education, work, and annual consumption level. Gender is classified as male and female. Age: under 20, 21–30, 31–40, 41–50, and over 50. Educational qualifications are classified by: high school and below, college, undergraduate, and postgraduate. Works are classified by civil servants, enterprise employees, students, freelancers, and others. The annual consumption level of personal travel is classified by below 500, 501–1,000, 1,001–2,000, 2,001–3,000, and over 3,000.

In this study, questionnaires were designed from five aspects: research purpose, tourists' experiential marketing perception, tourists' consumption emotion, tourists' revisit intention, and tourists' basic information. The survey part uses a Likert 7-dimension scale, with a score of 1 to 7 indicating satisfaction.

Each visitor who voluntarily answered the questionnaire was informed that the results of the questionnaire will be used in the data analysis and discussion of the results of this study. Meantime, after completing the questionnaire, they will get a coupon for Jinan cuisine as a reward. The first part introduces the purpose of the research to ensure the efficiency and reliability of filling (Rather et al., 2019). The second part investigates tourists' perception of experience (15 items). Questions 1–4 are perception items of sensory experience, questions 5–8 are perception items of action experience, questions 9–11 are perception items of emotional experience, and questions 12–15 are perception items of thinking experience. The third part investigates tourists' consumption emotion (4 items), that is, questions 16–19 are tourists' positive consumption emotion. The fourth part investigates tourists' revisit intention (4 items), that is, questions 20–23 are survey questions of tourists' revisit intention. The fifth part is the basic information consultation for tourists (five items), which asks the tourists' gender, age, education, work, and personal annual consumption level, respectively.

## Data Analysis and Empirical Testing

### Sample Characteristics

The questionnaires in this study were distributed in four places: Baotu Spring, Black Tiger Spring, Pearl Spring, and Wulong Pool (refer to section Case Study of Cultural Tourism City—Jinan City). A total of 382 questionnaires were distributed through “QQ Questionnaire Star” (an electronic questionnaire collection platform) in October 2021, and 305 valid questionnaires were retained after screening. The overall recovery rate of the questionnaire was 79.8%. Tourists can obtain electronic questionnaires by scanning the QR code at the entrance of the scenic spot and obtain sample data directly on the computer terminal after successful submission. The reliability and validity analysis results of the questionnaire are given in Tables A1, A2. This questionnaire is not only for Chinese people but also for tourists from all over the world. However, due to COVID-19, the respondents to the survey were all from China, of which 52.79% were tourists from Shandong Province, and the rest came from 33 other regions in China.

All methods were carried out in accordance with relevant guidelines and regulations. All research protocols were approved by Shandong Agricultural University Scientific Ethics Committee and Jinan University Science and Technology Ethics Committee. Consent was obtained from all subjects and/or their legal guardian(s). The datasets used and/or analyzed during the current study available from the corresponding author on reasonable request. The statistical distribution of the demographic characteristics of the sample data in this study is shown in Table 4.

**Table 4:** Statistics of demographic characteristics.

**Tourist demographics**	**Statistical description**	**Sample numbers**	**Percentage %**
Gender	Male	99	32.5
	Female	206	67.5
Age	<20	20	6.6
	21–30	76	24.9
	31–40	103	33.8
	41–50	94	30.8
	51–60	10	3.3
	>60	2	0.7
Education	High school and below	13	4.3
	College	58	19.0
	Undergraduate	154	50.5
	Postgraduate	80	26.2
Work	Civil servants	162	53.1
	Enterprise employees	46	15.1
	Students	34	11.1
	Free-lancers	24	7.9
	Others	39	12.8
Average annual tourism consumption of tourists	<500	9	3.0
	501–1,000	21	6.9
	1,001–2,000	48	15.7
	2,001–3,000	73	23.9
	>3,000	154	50.5

In terms of gender, the gender ratio of tourists in Jinan is disparate (male 32.5% vs. female 67.5%). In terms of age, there are more tourists aged 21–50 years (89.5%). Because parents of this age group have higher purchasing power, they are more willing to bring their children to Jinan to experience the cultural atmosphere of the cultural tourism city and gain cultural knowledge. In terms of educational background, the respondents with undergraduate are the most (50.5%), followed by postgraduate (26.2%). This is related to the fact that young respondents with higher education are more willing to accept questionnaires. In terms of occupational distribution, the proportion of civil servants is higher (53.1%), followed by enterprise employees (15.1%). It can be seen that the tourist crowd in Jinan is mainly from the government and public institutions. In terms of average annual tourism consumption of tourists, the majority (50.5%) have an annual consumption of more than 3,000 yuan, which shows that the overall consumption level of this survey is relatively high.

### Descriptive Statistics

Table 5 shows the results of descriptive statistical analysis of each item in the questionnaire, and the specific item scores are shown in Table A3 (scales 1–7). It can be seen from Table 5 that the average value of each question of tourism experience is around 5.0, indicating that tourist experience and positive consumption emotion are both high.

**Table 5:** Descriptive statistical analysis of each item.

	**Mean**	**Std. deviation**	**Skewness**	**Kurtosis**	**Alpha**
1. ***Jinan*** has unique architectural landscape	5.15	1.451	−0.623	0.103	0.844
2. ***Jinan*** has complete tourism facilities	5.1	1.47	−0.596	−0.043	
3. ***Jinan*** has convenient transportation	5.23	1.46	−0.832	0.365	
4. ***Jinan*** has good tourism services	5.04	1.546	−0.544	−0.398	
5. Traveling in ***Jinan*** is a matter of course	5.05	1.536	−0.51	−0.408	0.87
6. The service staff recommends me to try new things in ***Jinan***	5.67	1.402	−1.154	1.071	
7. ***Jinan*** provides enough consultation	5.43	1.408	−0.851	0.485	
8. Interact with folk craftsmen in ***Jinan***	5.49	1.379	−1.056	1.222	
9. ***Jinan*** makes me feel fresh and relaxed	5.21	1.396	−0.613	0.304	0.858
10. ***Jinan*** makes me feel warm and cordial	5.06	1.51	−0.603	0.038	
11. The atmosphere of ***Jinan*** makes me want to play	5.53	1.398	−1.072	1.13	
12. ***Jinan*** gives me a thought-provoking experience	5.33	1.387	−0.819	0.5	0.822
13. ***Jinan*** arouses my curiosity	5.65	1.361	−1.061	0.89	
14. Traveling in ***Jinan*** makes me resonate with “***Qilu Culture***”	5.4	1.38	−0.898	0.803	0.869
15. Traveling in ***Jinan*** gives me a high-quality sense of identity	5.47	1.449	−0.895	0.378	
16. I was surprised to play in ***Jinan***	5.01	1.414	−0.496	−0.246	0.93
17. I was delighted to play in ***Jinan***	5.42	1.291	−0.838	0.619	
18. ***Jinan*** enchanted me	5.45	1.282	−0.896	0.753	
19. ***Jinan*** impressed me	5.34	1.41	−0.863	0.502	
20. I will come back to ***Jinan*** soon	4.11	1.911	−0.135	−1.069	0.876
21. ***Jinan*** is my first choice for understanding “***Qilu Culture***”	3.93	1.79	0.097	−0.899	
22. I would recommend ***Jinan*** to anyone who wants to know about “***Qilu Culture***”	4.68	1.785	−0.422	−0.849	
23. I will encourage family and friends to visit ***Jinan***	5.11	1.725	−0.72	−0.406	

From the perspective of independent variable (tourism experience), about 70% of tourists are satisfied with Jinan's architectural landscape, tourist facilities, convenient transportation, tourism products, and services. Tourists have a high overall score on the action experience, and they are more satisfied with the existing action experience projects in Jinan. The emotional experience of tourists after playing is more balanced. The thinking experience that Jinan brings to tourists can resonate with tourists and gain their recognition. Most tourists believe that traveling to Jinan can make them have a high-quality thinking experience.

From the perspective of intermediate variable (consumption emotion), tourists have a high positive consumption emotion, and most tourists are surprised, delighted, enchanted, and impressed during the travel. From the perspective of tourists' revisit invention, compared with traveling to Jinan again, tourists are more willing to recommend to others.

From the perspective of dependent variable (revisit invention), it can also be seen from the data of tourists' revisit invention that the respondents have a good overall tourism experience in Jinan, and tourists are willing to strongly recommend it to others.

### Correlation Analysis

In this study, Spearman correlation is used to judge the relationship between tourist experience and consumption emotion, tourist experience and revisit intention, and consumption emotion and revisit intention (Table 6). The larger the absolute value of the correlation coefficient, the stronger the correlation, and vice versa. Correlation coefficient = 0.8–1.0 means very strong correlation, 0.6–0.8 means strong correlation, and 0.4–0.6 means medium correlation. The results in Table 6 show that the Spearman correlation *P*-value is <0.001, and the correlation coefficient between each dimension is almost within 0.6–0.8, indicating that there are statistically significant correlations between tourist experience and consumption emotion, Tourist experience and revisit intention, and consumption emotion and revisit intention. All six dimensions of sensory experience (SE), action experience (AE), emotional experience (EE), thinking experience (TE), consumption emotion (CE), and revisit intention (RI) all show a significant positive relationship.

**Table 6:** Correlation coefficients of dimensions in each group.

		**SE**	**AE**	**EE**	**TE**	**CE**	**RI**
SE	Correlation coefficient	1					
	Sig. (Bilateral)						
AE	Correlation coefficient	0.807	1				
	Sig. (Bilateral)	0.000					
EE	Correlation coefficient	0.716	0.780	1			
	Sig. (Bilateral)	0.000	0.000				
TE	Correlation coefficient	0.657	0.696	0.766	1		
	Sig. (Bilateral)	0.000	0.000	0.000			
CE	Correlation coefficient	0.711	0.748	0.789	0.820	1	
	Sig. (Bilateral)	0.000	0.000	0.000	0.000		
RI	Correlation coefficient	0.477	0.517	0.580	0.607	0.621	1
	Sig. (Bilateral)	0.000	0.000	0.000	0.000	0.000	

### Model Fitting and Test for Mediation

#### Fit Test of Original Model

**Figure 2:**
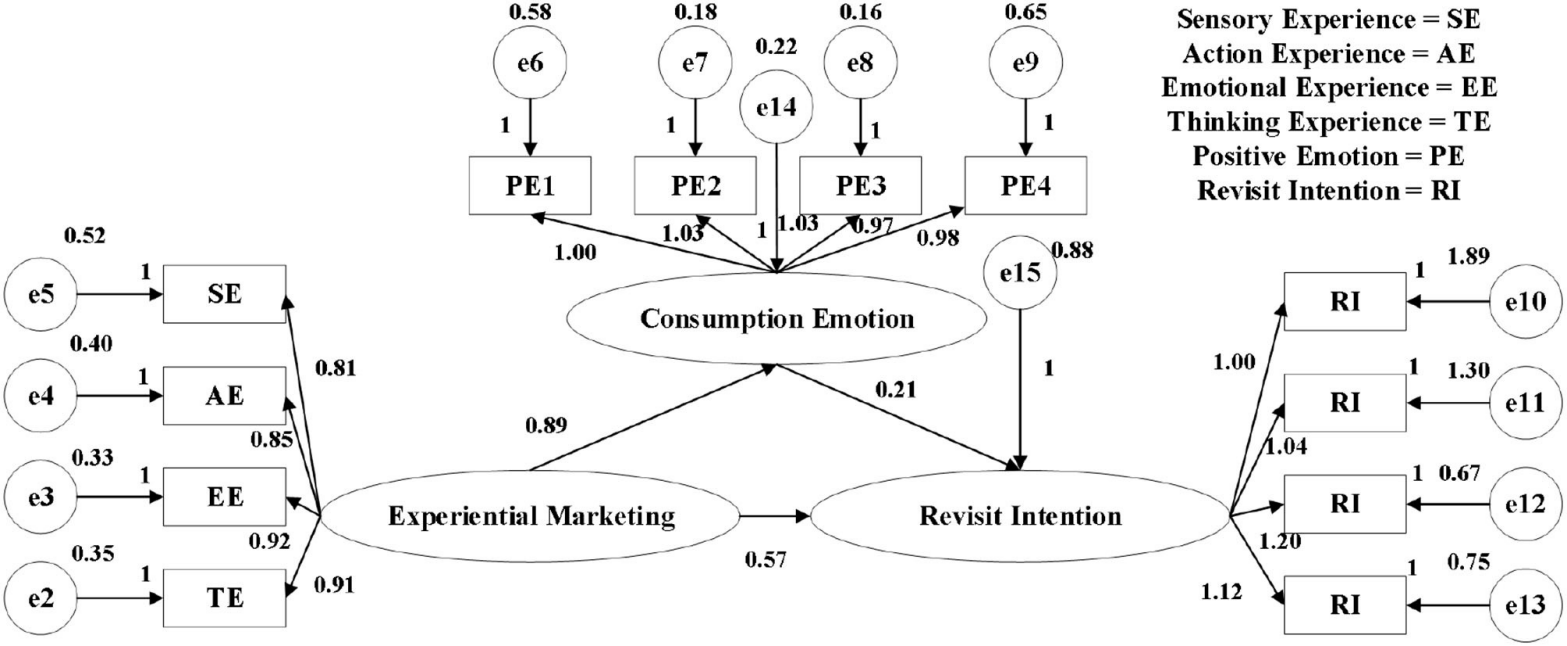
Initial model path diagram (normalized coefficients).

In this study, structural equation modeling was used to verify the causal relationship of the hypothesized model. In this study, AMOS software was used to analyze the fitting effect of the model, and it was found that the fitting situation before the correction was not good. When a factor model fits the data, factor loadings are chosen to minimize the difference between the correlation matrix implied by the model and the actual observed matrix. According to Table A4, the standardization coefficient factor loading values of the 13 observed variables in this study are between 0.683 and 0.950, and the number of error variables is between 0.098 and 0.520. In addition to the revisit willingness of <0.7, the rest of the factor load values in this study were all >0.7, which met the standard. The rest of the error variables are also <0.5. According to the recommended criteria of, CMIN/DF ≤ 3.0, RMR < 0.1, RMSEA ≤ 0.1, CFI ≥ 0.90, GFI ≥ 0.85, and NFI > 0.9. Table 7 indicates that the fitting degree of the model is acceptable. But from the data in Table 7 and Table A4, it can be seen that this model still has room for modification. RMR, NFI, and CFI all meet the criteria, but CMIN/DF, RMSEA, and GFI all fail. Therefore, the correction index MI is used to understand the reasons for the poor model fit. Figure 2 is the initial model of the study.

**Table 7:** Summary table of original model fits.

**Fit metrics**	**CMIN/DF**	**RMR**	**RMSEA**	**GFI**	**NFI**	**CFI**
Index value	4.449	0.077	0.107	0.875	0.932	0.946
Fit criteria	<3	<0.1	<0.080	>0.900	>0.900	>0.900
Fit evaluation	Non-compliant	Compliant	Non-compliant	Non-compliant	Compliant	Compliant

#### Analysis of Model Correction Process

In this study, AMOS computing software was used to analyze and interpret the model path. The MI correction index was added to the original model to improve the fit of the overall model by adding relevant paths, and the research model was revised according to the fitting results. In this study, the structural equation model was optimized by the MI correction index, and all residual terms with MI values >20 were connected by arrows to improve the fit of the overall structural equation. Under normal circumstances, only the maximum MI value is modified each time during modification, and the modification result is finally given. After the initial model optimization of the MI value, the research found that the indicators of the model were significantly improved.

#### Analysis of Model Correction Results

**Figure 3:**
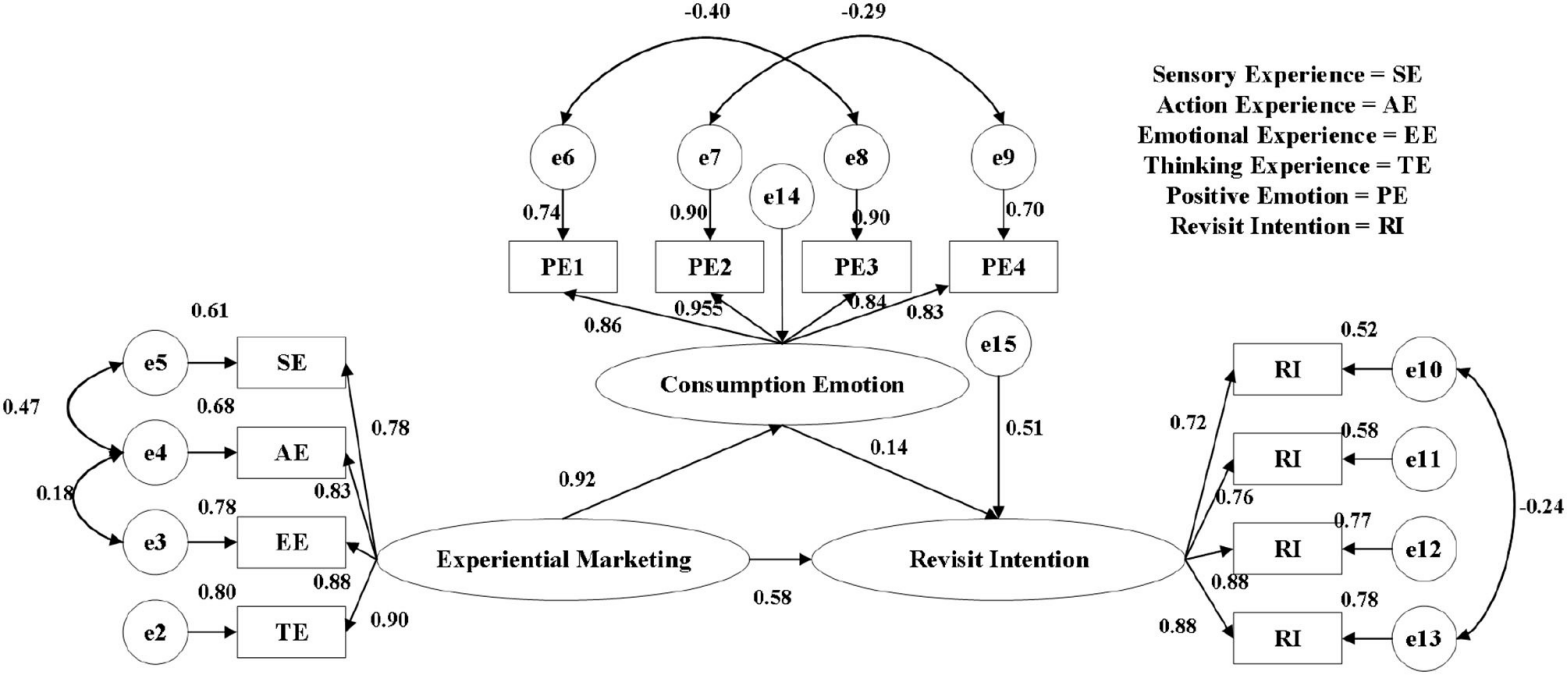
Modified model path diagram (normalization coefficient).

The parameter estimation results of the model in this study are shown in Table A5 and Table 8. From Table A5, each path has reached a significant level; from Table 8, each index has reached the model fitting requirements. Therefore, the fitting of the revised model is good, and the index values after the revision are all within the fitting standard range. The modified model fitting results are shown in Figure 3. It can be seen from Table 8 that the values of the model in this study are all within the standard range (CMIN/DF = 2.503, RMR = 0.064, RMSEA = 0.070, GFI = 0.935, NFI = 0.965, CFI = 0.978), which shows that the modified model is well-constructed. Therefore, it can be judged from the comprehensive evaluation index of the model revision and the reliability and validity analysis results that no further revision of the model is required.

**Table 8:** Comprehensive evaluation indicators of the revised model.

**Fit metrics**	**CMIN/DF**	**RMR**	**RMSEA**	**GFI**	**NFI**	**CFI**
Index value	2.503	0.064	0.070	0.935	0.965	0.978
Fit criteria	<3	<0.1	<0.080	>0.900	>0.900	>0.900
Fit evaluation	Compliant	Compliant	Compliant	Compliant	Compliant	Compliant

From the aforementioned analysis, it can be found that there is a good degree of fit between the hypothesis of this research model and the collected data, and the theoretical model can be accepted. The test results of each effect and hypothesis of the modified model path analysis are shown in Tables 9, 10. The analysis results are as follows:

(1) The relationship between experiential marketing and revisit invention

**Table 9:** Decomposition description of various effects of modified model path analysis.

			**Endogenous variable**			
			**Consumption emotion**		**Revisit invention**	
			**Normalized effect**	***t*** **-value**	**Normalized effect**	***t*** **-value**
Derived variable	Experiential marketing	Direct effect	0.919	19.812^*^	0.578	3.914^*^
		Indirect effect			0.142	0.995
		Overall effect	0.919	19.812^*^	0.720	4.909^*^
Endogenous variable	Consumption emotion	Direct effect			0.142	0.995
		Indirect effect				
		Overall effect			0.142	0.995

* *p < 0.001*.

**Table 10:** Summary table of empirical results of research hypotheses.

**Hypothesis**	**Path**	**Std. coefficient**	***t*-value**	**Test result**
***H*_1_**	Experiential Marketing → Revisit Invention	0.578^*^	3.914	Established
***H*_2_**	Experiential Marketing → Consumption Emotion	0.919^*^	19.812	Established
***H*_3_**	Consumption Emotion → Revisit Invention	0.142	0.995	Non-established
***H*_4_**	Experiential Marketing → Consumption Emotion → Revisit Invention	0.142	0.995	Non-established

* *Indicates that the larger the Std. Coefficient, the stronger the persuasiveness, that is, the hypothesis is established*.

From Tables 9, 10, it can be found that the standardized estimated value of experiential marketing on revisit invention is 0.58, and the *R*^2^ value is 0.4096, that is, the perception of experiential marketing can explain 40.96% of the variance of revisit invention, and *H*_1_ is established. The results show that tourists' perception of experiential marketing can significantly affect their revisit invention.

(2) Relationship between experiential marketing and consumption emotion

It can be found from Tables 9, 10 that the standardized estimated value of experiential marketing perception on consumption emotion is 0.92, and the *R*^2^ value is 0.8464, that is, experiential marketing perception can explain 85% of the variables of consumption emotion, and *H*_2_ is established. The results show that when tourists have more and better experience perception of *Jinan*, their consumption emotion will be more positive. In other words, tourists' perception of experiential marketing can significantly affect their consumption emotion.

(3) Relationship between consumption emotion and revisit intention

From Tables 9, 10, while controlling for experiential marketing, it can be found that the standardized estimated value of consumption emotion on revisit intention is 0.14, that is, the influence of consumption emotion on revisit intention does not reach a significant level, and *H*_3_ does not hold. When tourists have more positive consumption emotion perceptions of *Jinan* experiential marketing, it does not significantly affect their revisit intention. In other words, while controlling for experiential marketing, tourists' perception of consumption emotion cannot effectively predict their revisit intention.

Although Tables 9, 10 shows that while controlling for experiential marketing, there is a significant positive correlation between consumption emotion and revisit intention, it is found in the model that the predictive power of consumption emotion on revisit intention does not reach a significant level. The generation of consumption emotion is largely affected by personal psychological cognition, and external perception may not necessarily significantly affect personal consumption emotion.

(4) Mediating effect of consumption emotion

According to Tables 9, 10, the indirect effect of experiential marketing on revisit intention through consumption emotion does not reach a significant level (normalized effect: 0.142, *t*-value: 0.995), and *H*_4_ does not hold. The results show that experiential marketing perception does not indirectly influence revisit intention through consumption emotion. This result shows that consumption emotion does not have a mediating effect between experiential marketing and revisit intention.

## Discussion

Experiential marketing perception positively affects revisit intention. This study complements research on the impact of experiential marketing on post-tour behavior, providing empirical evidence for the association between experiential marketing and revisit intention. Tourists' revisit intention is also stronger after travel when tourists feel better about experiential marketing in cultural tourism cities, and their willingness to revisit cultural tourism cities will also increase.

Experiential marketing perception has a positive impact on consumption emotion (Drengner et al., 2008; Ge et al., 2021; Meilatinova, 2021). This study complements research on the significant relationship between experiential marketing and consumption emotion and also provides empirical evidence for a causal relationship between experiential marketing and consumption emotion (Brakus et al., 2009). When tourists feel better about experiential marketing in cultural tourism cities, their positive consumption emotion in cultural tourism cities will also be higher (Bigné et al., 2005). Therefore, experiential marketing activities can enhance the consumption emotion of tourists in cultural tourism blocks (Lo and Wu, 2014; Rather and Hollebeek, 2020; Rather et al., 2021). Jinan's catering should be standardized and hygienic, so that tourists' taste buds can perceive the characteristics of “Qilu culture.” To meet the eating habits of foreign tourists, Western restaurants and other restaurants that do not have traditional “Qilu characteristics” can consider transforming them into Western restaurants with “Qilu characteristics.” To meet the demand of Chinese and foreign tourists for afternoon tea, cafes can also add “Qilu characteristics,” from interior decoration to tableware, and even drink matching styles can be creatively reformed according to “Qilu characteristics,” making it different from the general cafe style. Coffee and pizza can also be an innovation. Of course, original intention is to convey more sensory experience, not just simple splicing on hardware devices.

The influence of consumption emotion on revisit intention is not significant, and the mediating effect of experiential marketing on revisit intention through consumption emotion is not significant (same as Derbaix and Pham, 1991; Ladhari, 2007; Rather, 2017; Lester et al., 2021). This study found that the mediating role played by tourists' consumption emotion in cultural tourism cities is not significant compared to that of tourism factories (Liu et al., 2021), when controlling for experiential marketing; is different from the mediating role played by experiential marketing in the retail industry (such as bicycle and car sales industry); and has no significant influence on revisit intentions (Han et al., 2010; Hou et al., 2013; Lee and Lee, 2016; Köchling, 2021).

The most likely reason for the difference in consumption emotion between the travel and retail industries is that consumers have different spending motivations (Gyte and Phelps, 1989; Bigné et al., 2005; Chung and Petrick, 2013; Marques et al., 2021). Although cultural tourism cities, like retail and tourism factories, do not charge tourists tickets, the purpose of customers or tourists is quite different. One is for the purpose of tourism experience, and the other is for the purpose of purchasing goods (Kozak, 2003; Rather, 2018). Tourists have differences in their revisit intentions because of differences in consumption motives (Rojas-De-Gracia and Alarcon-Urbistondo, 2019). For cultural tourism cities, the experience of tourists directly affects their intuitive feelings about tourism and its derivative products, and high-quality experience will also affect tourists' willingness to revisit and recommend (Hou et al., 2013).

### Research Gaps and Suggestions for Future Work

The effect of experiential marketing will have different research conclusions due to different industries. In the future work, we will further revise the scales of three dimensions of experiential marketing, consumption emotion, and revisit intention in this study. Future research should consider a comparative analysis of tourist experiential marketing in different cultural tourism cities; conduct research on the travel experience of different groups; find the impact of experiential marketing, consumption emotion, and revisit intention in a more targeted manner; focus more precisely on the individual consumption needs of tourists; and explore how experiential marketing affects the consumption emotion of specific tourist groups.

## Conclusion

This research selected the Chinese city of Jinan represented by “Qilu culture” as the research object; explored the relationship between experiential marketing, consumption emotion, and revisit intention; and used a structural equation model to verify the relationship among the three. This study divided the perception of experiential marketing into four dimensions—sensory experience, action experience, emotional experience, and thinking experience, and divided tourists' revisit intention into two dimensions—“revisit” and “recommendation.”

Under the background of experience economy, cultural tourism cities can enhance tourists' consumption emotion through experiential marketing, generate positive tourism consumption emotion, and then promote tourists to go to cultural tourism cities for secondary consumption. Although consumption emotion does not play a significant mediating role between experiential marketing and tourists' willingness to revisit, high-quality tourist consumption experience directly motivates tourists to revisit intention. Cultural tourism cities can start from Maslow's hierarchy of needs theory, pay attention to the needs of tourists at different levels, and adopt effective experiential marketing strategies from tourism experience to improve the quality of tourists' travel experience and promote tourists' revisit intention.

This study verifies the relationship model among experiential marketing, consumption emotion, and revisit intention through empirical research; enriches Schmitt's theory of the five modules of experiential marketing; and, to a certain extent, broadens the application field of experience economy in the consumption behavior of tourists in cultural tourism cities. At the same time, this study proposes a set of relational models about experiential marketing, consumption emotion, and revisit intention, providing a new perspective for tourism research. Finally, this research provides practical reference for the development of tourism products in other cultural tourism cities and helps guide the planning and development of tourism products, the design of tourism commodities, and the configuration of business management.

## Data Availability Statement

The raw data supporting the conclusions of this article will be made available by the authors, without undue reservation.

## Author Contributions

HC: resources, formal analysis, visualization, writing—review and editing, data curation, and formal analysis. YW: conceptualization, supervision, and writing—review and editing. NL: investigation, formal analysis, and supervision. All authors contributed to the article and approved the submitted version.

## Funding

This study was funded by the National Social Science Fund of China project *Research on the development path and strategy of Cultural and tourism integrated railway station area* (Grant No. 20CGL024).

## Conflict of Interest

The authors declare that the research was conducted in the absence of any commercial or financial relationships that could be construed as a potential conflict of interest.

## Publisher's Note

All claims expressed in this article are solely those of the authors and do not necessarily represent those of their affiliated organizations, or those of the publisher, the editors and the reviewers. Any product that may be evaluated in this article, or claim that may be made by its manufacturer, is not guaranteed or endorsed by the publisher.
