# Supplementary material for: Research on the Relationship of Consumption Emotion, Experiential Marketing, and Revisit Intention in Cultural Tourism Cities: A Case Study
Source: Front Psychol. 2022 Jul 13;13:894376. doi: 10.3389/fpsyg.2022.894376 (PMC9326360; doi:10.3389/fpsyg.2022.894376)
Supplement: Supplementary file 1 [file Data_Sheet_1.DOCX]

***Reliability Test***

This study uses Cronbach's Alpha coefficient to judge the reliability of the questionnaire. The larger the Cronbach's Alpha coefficient, the higher the internal reliability of the measurement variable. The overall Cronbach's Alpha coefficient of this study is 0.963, and the Cronbach's Alpha coefficient of each item is also above 0.7, and the CITC (Corrected Item - Total Correlation) value of each scale is greater than 0.4. This shows that the reliability of each measurement scale in the questionnaire is high. The reliability analysis results of each scale are shown in Table A1.

**Table A1. Reliability Analysis Results of Each Item**

| Measurement Item | Mean | Std. Deviation | CITC | Items Reliability | Alpha |
| --- | --- | --- | --- | --- | --- |
| 1. ***Jinan*** has unique architectural landscape | 5.16 | 1.434 | 0.664 | 0.962 | 0.844 |
| 2. ***Jinan*** has complete tourism facilities | 5.11 | 1.454 | 0.694 | 0.961 |  |
| 3. ***Jinan*** has convenient transportation | 5.24 | 1.442 | 0.620 | 0.962 |  |
| 4. ***Jinan*** has good tourism services | 5.05 | 1.531 | 0.679 | 0.962 |  |
| 5. Traveling in ***Jinan*** is a matter of course | 5.06 | 1.521 | 0.666 | 0.962 | 0.87 |
| 6. The service staff recommendes me to try new things in ***Jinan*** | 5.68 | 1.378 | 0.682 | 0.962 |  |
| 7. ***Jinan*** provides enough consultation | 5.44 | 1.387 | 0.749 | 0.961 |  |
| 8. Interact with folk craftsmen in ***Jinan*** | 5.50 | 1.357 | 0.789 | 0.961 |  |
| 9. ***Jinan*** makes me feel fresh and relaxed | 5.22 | 1.377 | 0.792 | 0.960 | 0.858 |
| 10. ***Jinan*** makes me feel warm and cordial | 5.07 | 1.494 | 0.774 | 0.961 |  |
| 11. The atmosphere of ***Jinan*** makes me want to play | 5.55 | 1.376 | 0.717 | 0.961 |  |
| 12. ***Jinan*** gives me a thought-provoking experience | 5.34 | 1.367 | 0.818 | 0.960 | 0.822 |
| 13. ***Jinan*** arouses my curiosity | 5.66 | 1.337 | 0.732 | 0.961 |  |
| 14. Traveling in ***Jinan*** makes me resonate with "***Qilu Culture***" | 5.41 | 1.359 | 0.845 | 0.960 | 0.869 |
| 15. Traveling in ***Jinan*** gives me a high-quality sense of identity | 5.49 | 1.428 | 0.805 | 0.960 |  |
| 16.I was surprised to play in ***Jinan*** | 5.02 | 1.398 | 0.791 | 0.960 | 0.93 |
| 17.I was delighted to play in ***Jinan*** | 5.43 | 1.267 | 0.846 | 0.960 |  |
| 18.***Jinan*** enchanted me | 5.46 | 1.258 | 0.840 | 0.960 |  |
| 19.***Jinan*** impressed me | 5.36 | 1.390 | 0.766 | 0.961 |  |
| 20.I will come back to ***Jinan*** soon | 4.12 | 1.906 | 0.512 | 0.964 | 0.876 |
| 21.***Jinan*** is my first choice for understanding "***Qilu Culture***" | 3.94 | 1.785 | 0.597 | 0.963 |  |
| 22.I would recommend ***Jinan*** to anyone who wants to know about "***Qilu Culture***" | 4.69 | 1.775 | 0.640 | 0.962 |  |
| 23.I will encourage family and friends to visit ***Jinan*** | 5.13 | 1.711 | 0.684 | 0.962 |  |

***CITC: Corrected Item - Total Correlation**

***Validity Analysis***

A total of 6 factors and 23 items were analyzed by Confirmatory Factor Analysis (CFA) in this study. Overall, each measurement item in this study showed significant statistical significance (***p***<0.001), and the Standardized Factor Loading (Std. FL) were all greater than 0.7 (Table A2). This shows that there is a good correspondence between factors and measurement items, and the convergent validity is good, which is suitable for factor analysis. The actual sample size of this analysis is 305, which is 10 times more than the number of analysis items, and the sample size is moderate. It can be seen from Table A2 that the AVE values corresponding to the six factors are all greater than 0.5, and the CR values are all higher than 0.7, which means that the data in this analysis have good convergent validity.

**Table A2. Factor Loading Factor Table**

| Factor  (Latent Variable) | Analysis Term (Explicit Variable) | Coef. | Std.  Error | ***z****-Test* | ***p****-Value* | Std. FL | AVE Value | CR Value |
| --- | --- | --- | --- | --- | --- | --- | --- | --- |
| Sensory Experience | 1. ***Jinan*** has unique architectural landscape | 1.000 | - | - | - | 0.763 | 0.576 | 0.844 |
|  | 2. ***Jinan*** has complete tourism facilities | 1.049 | 0.074 | 14.095 | 0.000 | 0.790 |  |  |
|  | 3. ***Jinan*** has convenient transportation | 0.963 | 0.075 | 12.924 | 0.000 | 0.731 |  |  |
|  | 4. ***Jinan*** has good tourism services | 1.050 | 0.079 | 13.304 | 0.000 | 0.750 |  |  |
| Action Experience | 5. Traveling in ***Jinan*** is a matter of course | 1.000 | - | - | - | 0.715 | 0.621 | 0.868 |
|  | 6. The service staff recommendes me to try new things in ***Jinan*** | 0.991 | 0.075 | 13.265 | 0.000 | 0.782 |  |  |
|  | 7. ***Jinan*** provides enough consultation | 1.045 | 0.075 | 13.913 | 0.000 | 0.820 |  |  |
|  | 8. Interact with folk craftsmen in ***Jinan*** | 1.055 | 0.074 | 14.355 | 0.000 | 0.846 |  |  |
| Emotional Experience | 9. ***Jinan*** makes me feel fresh and relaxed | 1.000 | - | - | - | 0.845 | 0.664 | 0.855 |
|  | 10. ***Jinan*** makes me feel warm and cordial | 1.042 | 0.060 | 17.355 | 0.000 | 0.811 |  |  |
|  | 11. The atmosphere of ***Jinan*** makes me want to play | 0.931 | 0.056 | 16.555 | 0.000 | 0.787 |  |  |
| Thinking Experience | 12. ***Jinan*** gives me a thought-provoking experience | 1.000 | - | - | - | 0.879 | 0.704 | 0.825 |
|  | 13. ***Jinan*** arouses my curiosity | 0.884 | 0.049 | 17.905 | 0.000 | 0.795 |  |  |
|  | 14. Traveling in ***Jinan*** makes me resonate with "***Qilu Culture***" | 1.000 | - | - | - | 0.903 |  |  |
|  | 15. Traveling in ***Jinan*** gives me a high-quality sense of identity | 0.991 | 0.046 | 21.587 | 0.000 | 0.851 |  |  |
| Consumption Emotion | 16.I was surprised to play in ***Jinan*** | 1.000 | - | - | - | 0.837 | 0.777 | 0.933 |
|  | 17.I was delighted to play in ***Jinan*** | 1.026 | 0.045 | 22.97 | 0.000 | 0.947 |  |  |
|  | 18.***Jinan*** enchanted me | 1.011 | 0.045 | 22.658 | 0.000 | 0.940 |  |  |
|  | 19.***Jinan*** impressed me | 0.969 | 0.055 | 17.656 | 0.000 | 0.816 |  |  |
| Revisit Intention | 20.I will come back to ***Jinan*** soon | 1.000 | - | - | - | 0.689 | 0.640 | 0.876 |
|  | 21.***Jinan*** is my first choice for understanding "***Qilu Culture***" | 1.043 | 0.085 | 12.199 | 0.000 | 0.767 |  |  |
|  | 22.I would recommend ***Jinan*** to anyone who wants to know about "***Qilu Culture***" | 1.197 | 0.087 | 13.752 | 0.000 | 0.886 |  |  |
|  | 23.I will encourage family and friends to visit ***Jinan*** | 1.124 | 0.083 | 13.491 | 0.000 | 0.862 |  |  |

***Coef. = Non-standard load coefficient; Std. Error = Standard Error; *z*-Test = Test for Difference of Means; *p*-Value = statistical significance; Std. FL = Standardized Factor Loading; AVE Value = Average Variance Extracted; CR Value = Composite Reliability**

***Results of the Questionnaire***

**Table A3. Description of scale**

|  |  |  | Percentage of Different Scores (%) | | | | | | |
| --- | --- | --- | --- | --- | --- | --- | --- | --- | --- |
|  |  |  | 1 | 2 | 3 | 4 | 5 | 6 | 7 |
| Experiential  Marketing | Sensory  Experience | Architectural Landscape | 1.6 | 3.6 | 5.9 | 18 | 28.9 | 20.3 | 21.6 |
|  |  | Tourist Facilities | 1.6 | 3.9 | 7.2 | 18.4 | 27.2 | 21.3 | 20.3 |
|  |  | Convenient Transportation | 1.6 | 4.6 | 5.2 | 13.1 | 28.5 | 25.6 | 21.3 |
|  |  | Tourism Products and Services | 1.6 | 4.9 | 10.8 | 15.1 | 24.9 | 22 | 20.7 |
|  | Action  Experience | Travel for Granted | 1.6 | 4.3 | 10.5 | 17.4 | 23.6 | 21.3 | 21.3 |
|  |  | Try New Things | 1.3 | 2 | 4.3 | 10.5 | 19 | 27.5 | 35.4 |
|  |  | Consumption | 1.3 | 2.3 | 4.9 | 13.8 | 25.6 | 24.3 | 27.9 |
|  |  | Interaction | 2 | 1.6 | 3.3 | 12.8 | 25.2 | 28.2 | 26.9 |
|  | Emotional  Experience | Relaxed and Comfortable | 1.6 | 2 | 5.6 | 18 | 31.5 | 18.7 | 22.6 |
|  |  | Warm, Cordial | 3 | 2.3 | 7.2 | 22 | 23.6 | 21.3 | 20.7 |
|  |  | Desire to Play | 2 | 1.3 | 4.3 | 11.8 | 23.3 | 27.9 | 29.5 |
|  | Thinking  Experience | Thought Provoking | 1.3 | 2.6 | 5.2 | 14.4 | 26.9 | 26.9 | 22.6 |
|  |  | Curiosity | 0.7 | 2.6 | 4.3 | 8.5 | 23.9 | 25.9 | 34.1 |
|  |  | Related to "***Qilu Culture***" | 1.6 | 2 | 4.3 | 13.8 | 27.5 | 25.9 | 24.9 |
|  |  | High-grade Recognition | 1.3 | 2.3 | 6.9 | 10.5 | 24.9 | 23.3 | 30.8 |
| Consumption  Emotion |  | Surprised | 1 | 3.6 | 10.5 | 17.7 | 27.2 | 24.6 | 15.4 |
|  |  | Delighted | 0.7 | 1.6 | 5.9 | 11.8 | 27.2 | 31.1 | 21.6 |
|  |  | Enchanted | 0.7 | 1.6 | 5.9 | 11.8 | 27.2 | 31.1 | 21.6 |
|  |  | Impressed | 1.6 | 2 | 6.6 | 13.8 | 23.9 | 29.2 | 23 |
| Revisit  Invention |  | Travel to ***Jinan*** again | 12.8 | 10.9 | 12.5 | 19.7 | 16.1 | 15.1 | 12.8 |
|  | First choice for understanding "***Qilu Culture***" | | 9.9 | 14.1 | 16.8 | 23 | 14.5 | 10.9 | 10.9 |
|  |  | Inform Friends and Family | 5.3 | 8.6 | 13.5 | 14.5 | 18.8 | 21.4 | 18.1 |
|  |  | Recommend to People | 4.3 | 3.6 | 12.5 | 11.8 | 17.1 | 24 | 26.6 |

***Factor Load Analysis***

Used statistical parameters are: Goodness of Fit Index (GFI), Chi-square Fit Index (*x*), Root Mean Square of Estimated Error (RMSEA) and Comparative Fit Index (CFI). If RMSEA is less than or equal to 0.05, the model fits well. If the RMSEA is less than 0.08, there is an appropriate model fit. Strict is less than 0.05, not strict is less than 0.1, NFI is the normative fit index, which varies between 0 and 1, with 1=complete fit. By convention, Normative Fit Index (NFI) less than 0.90 indicates that the model needs to be reset. GFI is a goodness-of-fit index, which ranges between 0 and 1, but can theoretically produce meaningless negative numbers. As specified, to accept the model, the GFI should be equal to or greater than 0.90. CFI is the comparative fit index, which has a value between 0 and 1. A CFI close to 1 indicates a very good fit, and a value greater than 0.90 indicates an acceptable model. According to the recommended criteria of Bentler (1990), CMIN/DF≤3.0, CFI≥0.90, GFI≥0.85, RMSEA≤0.1, it indicates that the fitting degree of the model is acceptable.

**Table A4. Analysis Results of Original Model**

| Factor Construct | Measurement Standard | Factor Loading | Reliability Coefficient | Measurement Error |
| --- | --- | --- | --- | --- |
| Experiential Marketing | Sensory Experience | 0.809 | 0.654 | 0.346 |
|  | Action Experience | 0.856 | 0.733 | 0.267 |
|  | Emotional Experience | 0.892 | 0.796 | 0.204 |
|  | Thinking Experience | 0.883 | 0.780 | 0.220 |
| Consumption Emotion | Surprised | 0.842 | 0.709 | 0.291 |
|  | Delighted | 0.950 | 0.903 | 0.098 |
|  | Enchanted | 0.942 | 0.887 | 0.113 |
|  | Impressed | 0.821 | 0.674 | 0.326 |
| Revisit Invention | Revisit soon | 0.683 | 0.480 | 0.520 |
|  | First choice | 0.769 | 0.591 | 0.409 |
|  | Inform family and friends | 0.889 | 0.790 | 0.210 |
|  | Recommend to others | 0.863 | 0.745 | 0.255 |
| Adaptation Standard Value | | >0.700 | >0.500 | <0.500 |

***Analysis of Model Correction Results***

**Table A5. Summary Table of Regression Coefficients of Modified Model**

|  | Estimate | S.E. | C.R. | P | Label |
| --- | --- | --- | --- | --- | --- |
| Consumption Emotion ← Experiential Marketing | 0.920 | 0.045 | 19.812 | *** | par_1 |
| Revisit Invention ← Consumption Emotion | 0.140 | 0.162 | 0.995 | 0.320 | par_2 |
| Revisit Invention ← Experiential Marketing | 0.580 | 0.165 | 3.914 | *** | par_3 |
| Surprised ← Consumption Emotion | 1.000 |  |  |  |  |
| Delighted ← Consumption Emotion | 1.010 | 0.042 | 24.224 | *** | par_4 |
| Enchanted ← Consumption Emotion | 1.002 | 0.047 | 21.512 | *** | par_5 |
| Impressed ← Consumption Emotion | 0.968 | 0.052 | 18.521 | *** | par_6 |
| Revisit ← Revisit Invention | 1.000 |  |  |  |  |
| First Choice ← Revisit Invention | 0.987 | 0.079 | 12.443 | *** | par_7 |
| Inform ← Revisit Invention | 1.133 | 0.081 | 14.040 | *** | par_8 |
| Recommendation ← Revisit Invention | 1.103 | 0.083 | 13.330 | *** | par_9 |
| Thinking Experience ← Experiential Marketing | 0.918 | 0.035 | 26.437 | *** | par_10 |
| Emotional Experience←Experiential Marketing | 0.904 | 0.036 | 25.106 | *** | par_11 |
| Action Experience←Experiential Marketing | 0.812 | 0.038 | 21.298 | *** | par_12 |
| Sensory Experience ← Experiential Marketing | 0.774 | 0.041 | 18.829 | *** | par_13 |
